# Prevalence and characteristics of prenatal cannabis use in Michigan, USA: A statewide population‐based pregnancy cohort

**DOI:** 10.1111/add.70188

**Published:** 2025-09-15

**Authors:** Ban Al‐Sahab, Jean M. Kerver, Omayma Alshaarawy, Kipling M. Bohnert, Michael R. Elliott, Hongxiang Qiu, Audriyana Jaber, Harish Neelam, Nigel Paneth

**Affiliations:** ^1^ Department of Family Medicine Michigan State University East Lansing MI USA; ^2^ Department of Epidemiology & Biostatistics Michigan State University East Lansing MI USA; ^3^ Department of Pediatrics and Human Development Michigan State University East Lansing MI USA; ^4^ Department of Biostatistics University of Michigan Ann Arbor MI USA

**Keywords:** cannabis, characteristics, marijuana, Michigan, pregnancy, prenatal, prevalence

## Abstract

**Aims:**

To estimate the prevalence of prenatal cannabis use in Michigan, USA, using self‐reports and urine toxicology and to examine factors associated with use.

**Design:**

Observational study using data from the Michigan Archive for Research on Child Health (MARCH) cohort, part of the National Institutes of Health's Environmental influences on Child Health Outcomes (ECHO) program. It is a prospective statewide pregnancy cohort recruited in 2017–2023 using births as a sampling frame.

**Setting:**

A three‐stage stratified cluster sample design that randomly selected hospitals, prenatal clinics and mothers across the lower peninsula of Michigan, where 97% of Michigan hospital births take place.

**Participants:**

Pregnant individuals recruited at their first prenatal visit primarily from 20 clinics serving 11 hospitals. Of 1105 eligible pregnant participants, 1092 (98.8%) had information on cannabis use either from self‐reports (*n* = 999), urine toxicology (*n* = 1028) or both (*n* = 945). Study participants had a median age of 29.3 years (interquartile range = 7.6).

**Measurements:**

Cannabis use was self‐reported by interviewer‐administered phone surveys twice during pregnancy and further ascertained by measuring tetrahydrocannabinol (THC) metabolites in up to two urine samples collected during pregnancy. The prevalence of cannabis use was estimated using sampling weights and clustering by sampled hospital to account for the complex survey design.

**Findings:**

The weighted prevalence of prenatal cannabis use was 16.8% [95% confidence interval (CI) = 8.0%–25.7%] based on either self‐report or urinalysis. Prevalence based on self‐report alone was 12.3% (95% CI = 6.1%–18.6%) and from urinalysis alone was 13.3% (95% CI = 5.1%–21.6%). Adjusted for creatinine concentrations, levels of 11‐nor‐9‐carboxy‐tetrahydrocannabinol (THC‐COOH) in positive samples ranged from 5.43 to 4694.89 ng/mg with a median of 122.00 ng/mg. Multivariate analysis revealed that participants who were single [adjusted prevalence rate ratio (aPRR) = 2.08, 95% CI = 1.15–3.78], had lower educational attainment (aPRR = 3.76, 95% CI = 1.23–11.53 for some college education and aPRR = 3.15, 95% CI = 0.97–10.25 for high school education as compared with undergraduate education or above), reported symptoms of depression (aPRR = 1.72, 95% CI = 1.19–2.48) and had a history of adverse childhood events (ACEs; aPRR = 2.04, 95% CI = 1.21–3.45 for ACEs ≥ 3 and aPRR = 1.64, 95% CI = 1.07–2.52 for 1–2 ACEs as compared to 0 ACEs) were more likely to use cannabis prenatally.

**Conclusions:**

Cannabis use is estimated to have occurred in one of six pregnancies in a population‐based sample in Michigan, USA.

## INTRODUCTION

As more US jurisdictions legalize cannabis, it is increasingly being used during pregnancy for both medical and recreational reasons [1, 2, 3]. From 2002 to 2017, self‐reported cannabis in pregnancy rose in the USA from 3.4% to 7.0%, respectively [1]. The legalization process has apparently changed perceptions of cannabis safety in pregnancy [4, 5, 6]. Between 2005 and 2015, public perception that the regular use of cannabis was harmless in pregnancy increased from 3.5% to 16.5% in pregnant individuals who do not use cannabis, and from 25.8% to 65.4% in pregnant individuals who currently use cannabis [4]. In addition, cannabis is now more potent. The average concentration of tetrahydrocannabinol (THC), the main psychoactive constituent of cannabis, increased from 10% in 2009 to 14% in 2019 [7].

Tetrahydrocannabinol (THC) crosses the placenta and can harm placental function [8, 9, 10]. Growing evidence suggests that cannabis exposure *in utero* may be associated with fetal growth restriction and preterm birth [11, 12, 13, 14, 15]. In some, but not all, studies, prenatal cannabis use is associated with attention deficit hyperactivity disorder (ADHD) symptoms, autism spectrum disorder and other neurodevelopmental outcomes [16, 17, 18, 19, 20]. Emerging data also show adverse effects of cannabis use on maternal pregnancy health [21].

As of April 2025, 39 states and the District of Columbia (DC) have legalized medicinal cannabis use, and 24 states and DC have legalized adult recreational use [22]. In Michigan, cannabis was legalized for medical use in 2008 and for adult recreational use in 2018, becoming the first Midwestern state to permit recreational use [22]. The changing legal and social landscape around cannabis requires better understanding of trends and correlates of cannabis use. Self‐reports likely underestimate use owing to social desirability bias, recall bias, and fear of discrimination and legal consequences [23, 24]. A study from a large healthcare system in Northern California in 2009–2017 revealed that the prevalence of prenatal cannabis use was twice as high when based on urine toxicology (4.9%) than when based on self‐report (2.5%) [23]. Similarly, only 6% of a sample of women delivering in Colorado in 2016 self‐reported past‐month use, but as many as 22.4% tested positive for cannabis based on umbilical cord assays [25].

The goal of this study was to: (i) quantify, using validated measures, the prevalence of cannabis use during pregnancy in Michigan; and (ii) examine its associated characteristics. The study is unique in estimating the statewide prevalence of prenatal cannabis use using both self‐reported data and urine toxicology, providing findings that are generalizable to the pregnant population of an entire state.

## METHODS

### Study design

The Michigan Archive for Research on Child Health (MARCH) cohort, part of the National Institutes of Health Environmental influences on Child Health Outcomes (ECHO) program, is a prospective statewide pregnancy cohort using births as a sampling frame. The MARCH cohort is based on a three‐stage stratified probability‐based sampling design, as described in detail elsewhere (Appendix S1). Briefly, in the first stage, a stratified random sample of 10 of the 95 hospitals with maternity services in the lower peninsula of Michigan was selected. While our sample is technically representative of the lower peninsula of Michigan, 97% of Michigan hospital births are in the lower peninsula [26, 27]. Thus, we refer to Michigan throughout the manuscript. In the second stage, two prenatal clinics referring to the 10 sampled hospitals were randomly sampled, weighted by number of births to clinic patients at the sampled hospital. One hospital, in Flint, MI, was added because of a history of regional environmental contamination. Overall, recruitment was primarily conducted in 20 clinics serving 11 hospitals across the state, with partial recruitment from 2 additional clinics, between 2017 and 2023. For the third stage of sampling, a target of 50 births was set for each sampled clinic. Sampling weights were constructed to account for the complex survey design and the multi‐stage sampling of the hospitals (Appendix S1).

The MARCH data were collected from questionnaires, medical records, birth certificate data and biological samples during pregnancy. For the present study, we used interviewer‐administered telephone surveys conducted twice during pregnancy [the median gestational age (GA) was 14 and 33 weeks for the prenatal 1 and prenatal 2 surveys, respectively] and urine samples collected up to three times (the median GA was 12, 27 and 33 weeks for visits 1, 2 and 3, respectively). Urine specimens were aliquoted into multiple samples and stored at −80°C in a monitored biobank and analyzed for this study between June 2022 and November 2023.

This study was approved by the Michigan State University (MSU) Institutional Review Board (IRB) [28] and follows the Strengthening the Reporting of Observational Studies in Epidemiology (STROBE) guidelines.

### Study participants

The MARCH cohort enrolled 1105 consenting English‐speaking participants who were at least 18 years of age at their first prenatal visit in the sampled clinics, who delivered 1130 babies in the sampled hospitals. Prenatal cannabis status was not confirmed in 13 pregnancies owing to missing interview data in addition to missing urine samples (*n* = 10), insufficient urine volume (*n* = 2) or a urine creatinine level of <0.2 mg/mL [29], which is inconsistent with human levels (*n* = 1). A total of 1092 participants were included in this analysis (Figure S1).

### Outcome

Prenatal cannabis use was defined as self‐reported in the first or second prenatal survey, and/or as a positive urine test on any urine sample. Self‐reported use was based on an affirmative answer to the question ‘*have you used marijuana (pot) or cannabis, including medically prescribed cannabis, at all during this pregnancy*’ on either prenatal survey. Participants were also asked about cannabis use in the 3 months prior to pregnancy. To confirm urine positivity, we analyzed two samples – from visit 1 and from visit 3. If either or both samples were absent, we examined the visit 2 sample. Urine samples were screened for 11‐nor‐9‐carboxy‐delta‐9‐THC (THC‐COOH) using qualitative enzyme‐linked immunosorbent assay (I‐50 sensitivity = 0.5 ng/mL; Neogen, Lansing, MI, USA). Samples screening positive on the Tecan Infinite M1000 plate reader (Tecan, Männedorf, Switzerland) underwent quantification by liquid chromatography with tandem mass spectrometry (LC/MS). Urine samples were analyzed for THC‐COOH (ng/mL) and creatinine (mg/mL) concentrations to serve as an indicator of urine dilution. Prenatal cannabis use was considered positive if THC metabolites were detected above the lower limits of detection (15 ng/mL) [29].

### Covariates

Covariates, obtained in the first prenatal survey, were maternal age, marital status, annual household income, highest level of educational attainment, race and ethnicity, enrollment in a government health insurance plan, pregnancy intention, symptoms of depression, social support, adverse childhood experiences (ACEs), experiences of discrimination, prenatal tobacco smoking and prenatal alcohol use. Symptoms of depression were measured using the 10‐item Edinburgh depression scale [30]. Any participant scoring 12 or more on this 30‐point scale (3 points per item) was considered to have experienced symptoms of depression [30]. The social support scale was composed of 14 items [31] and we examined scores above and below the median value of the scale. Discrimination was assessed using a modified version of the Experiences of Discrimination (EOD) scale [32]. ACEs were measured with seven items covering traumatic events experienced before the age of 13 years [33].

### Statistical analysis

The prevalence of cannabis use was estimated using sampling weights and clustering by sampled hospital. We present the weighted prevalence of cannabis use by ascertainment method, geographical region and timing of urine collection. We used Poisson regression with a log‐link function and model‐robust standard errors to estimate the prevalence rate ratio (PRR) of prenatal cannabis use and 95% confidence intervals (95% CIs). Clustering, however, was ignored in the multivariate regression models because of the limited degrees of freedom for variance estimation. All parameter estimates with two‐sided *P* < 0.05 were considered significant. Data cleaning and management were conducted using the Statistical Package for Social Sciences (SPSS 29.0; IBM, Armonk, NY, USA) and analyses were performed in R 4.4.0 (R Foundation for Statistical Computing, Vienna, Austria). This analysis was not pre‐registered, and the results should therefore be considered exploratory.

## RESULTS

**FIGURE 1:**
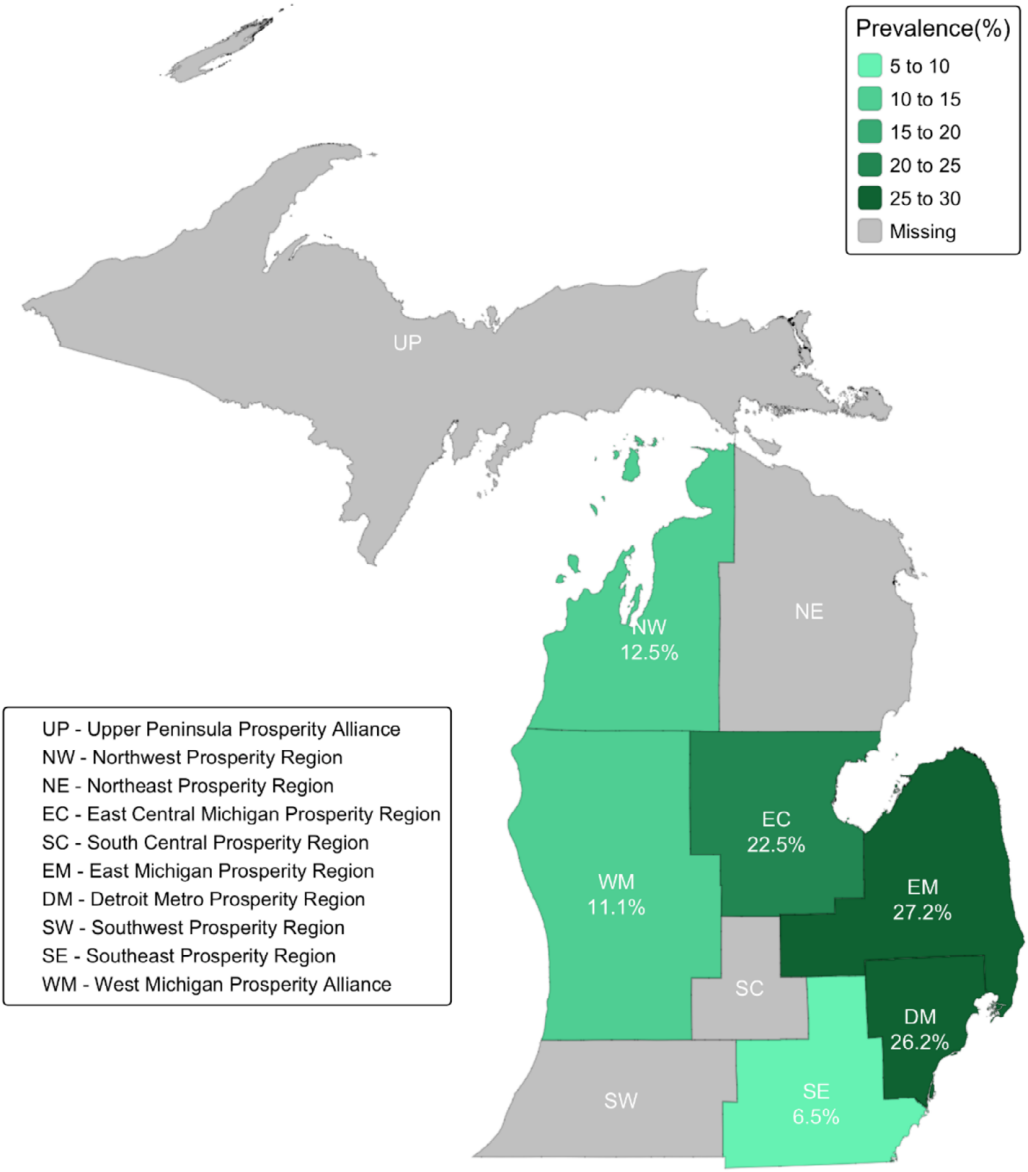
Weighted prevalence of prenatal cannabis use by geographic regions of sampled hospitals in Michigan, 2017–2023.

Of the 1092 participants, 945 participants (86.5%) had both cannabis assessments available, while 54 participants (4.9%) had data from self‐reports only and 93 participants (8.5%) had data from urine toxicology only. Urine toxicology testing was completed for 1875 urine samples from 1038 participants; 837 participants (80.6%) had two urine samples and 201 participants (19.4%) had only one urine sample. Table 1 presents the weighted prevalence of cannabis use by the different ascertainment methods. The overall weighted prevalence of cannabis use from self‐reports and/or urine toxicology was 16.8% (95% CI = 8.0%–25.7%). Of the participants who had used cannabis during pregnancy, 24.5% (95% CI = 10.6%–38.5%) were identified by self‐report only, 31.8% (95% CI = 17.0%–46.6%) were identified by urine toxicology only and 43.7% (95% CI = 31.9%–55.4%) were identified by both. Figure 1 represents the geographical variation in the prevalence of prenatal cannabis use by prosperity regions (i.e. a geographical framework created by Michigan’s Regional Prosperity Initiative) of the selected hospitals, which were situated in four of the seven lower peninsula prosperity regions. The weighted prevalence was highest in the East Michigan prosperity region (27.2%), followed by Detroit Metro (26.2%) and East Central Michigan (22.5%). The lowest prevalence was detected in the Southeast prosperity region (6.5%).

**TABLE 1:** Prevalence of cannabis use by ascertainment method among pregnant individuals in Michigan, 2017–2023.

Ascertainment method	Total	Prenatal cannabis use		THC‐COOH level (ng/mg creatinine)
	*n* ^a^	*n* ^a^	Weighted % (95% CI)	Median (IQR)
Self‐report				
Before pregnancy^b^	998	306	25.5% (17.0%–34.0%)	–
During pregnancy	999	162	12.3% (6.1%–18.6%)	–
Urine toxicology	1038	212	13.3% (5.1%–21.6%)	122.00 (207.70)
Visit 1^c^	998	198	13.0% (4.5%–21.5%)	117.15 (180.65)
Visit 2^c^	194	22	7.4% (1.8%–13.0%)	73.72 (603.60)
Visit 3^c^	683	71	6.3% (2.1%–10.5%)	130.15 (340.19)
Self‐report or urine toxicology	1092	260	16.8% (8.0%–25.7%)	–

^a^ Unweighted sample size.

^b^ Participants self‐reported cannabis use in the 3‐month period before pregnancy.

^c^ Corresponds to the prenatal study visit where the urine sample was collected.

The weighted prevalence of self‐reported cannabis use was 25.5% (95% CI = 17.0%–34.0%, *n* = 998) in the 3 months prior to pregnancy and 12.3% (95% CI = 6.1%–18.6%, *n* = 999) during pregnancy. All but three participants who reported cannabis use during pregnancy also reported cannabis use in the 3 months before pregnancy.

**FIGURE 2:**
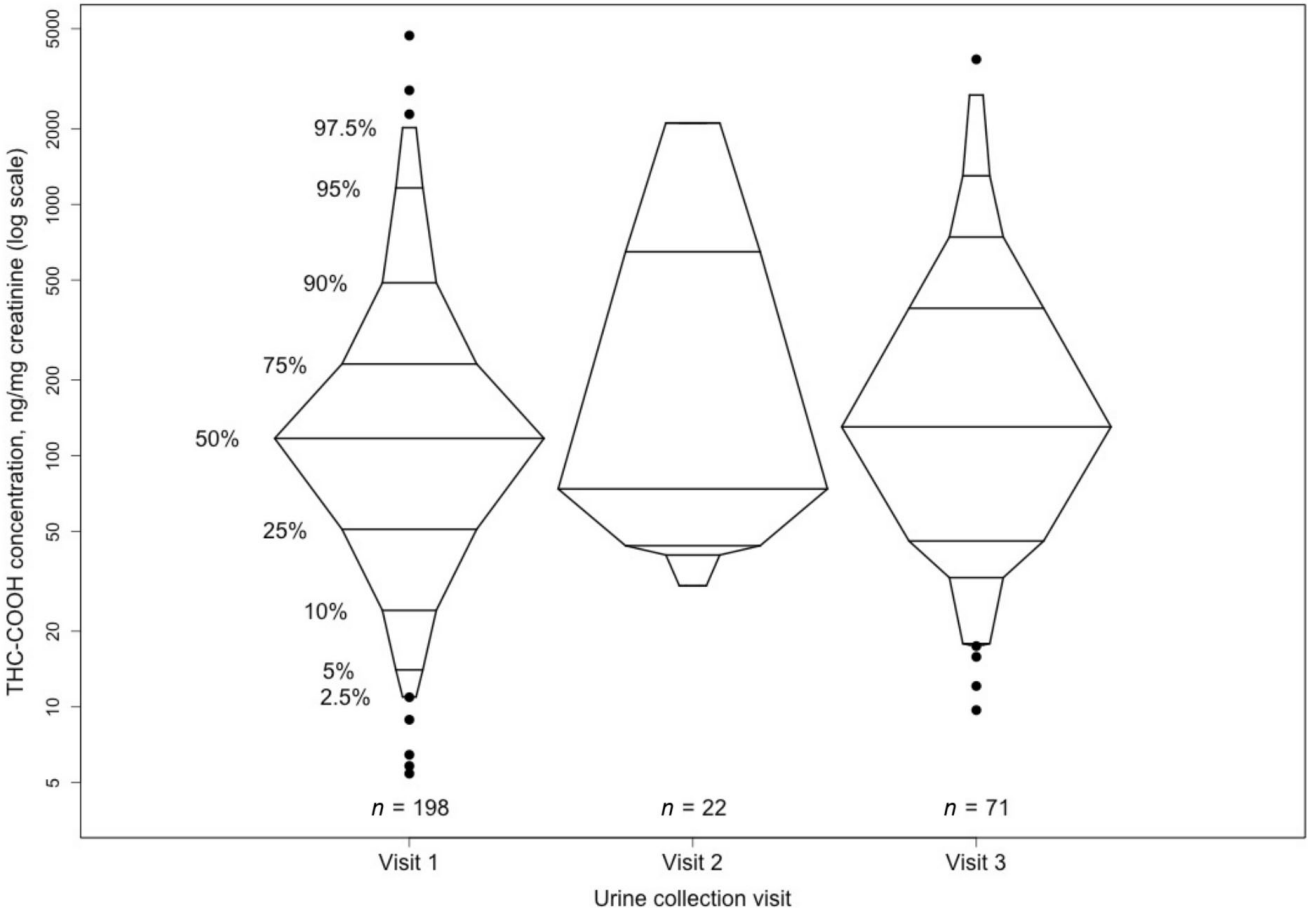
Creatinine‐normalized THC‐COOH concentrations by urine collection visit for positive urine samples, Michigan, 2017–2023.

Based solely on urinalysis results, the weighted prevalence of prenatal cannabis use was 13.3% (95% CI = 5.1%–21.6%). It was highest in visit 1 (13.0%, 95% CI = 4.5%–21.5%) and lowest in visit 3 (6.3%, 95% CI = 2.1%–10.5%). Among participants who tested positive on urine toxicology and had more than one urine toxicology test (*n* = 147), more than half tested positive twice (51.6%). The range of THC‐COOH (adjusted for creatinine concentrations) in urine‐positive samples ranged from 5.43 to 4694.89 ng/mg, with a median of 122.0 ng/mg (IQR = 207.7 ng/mg). Creatinine‐normalized THC‐COOH concentrations by specific visit are shown in Figure 2. Because urine was collected at routine prenatal care visits, GA at collection varied among study participants. The weighted prevalences of use for the first, second and third trimesters were 12.7% (95% CI = 3.3%–22.0%), 11.6% (95% CI = 1.3%–21.9%) and 7.1% (95% CI = 3.2%–11.1%), respectively.

Table 2 summarizes the prevalence rates and prevalence risk ratios of cannabis use during pregnancy by socio‐demographic status, psychological factors, and in relation to other substance use. Participants who were younger, unmarried, had lower educational attainment, had lower income, or were enrolled in a governmental health insurance plan were more likely to use cannabis, as were participants with unplanned pregnancies, with depressive symptoms, and with lower social support. Experience of ACEs and discrimination events were also associated with prenatal cannabis use. Cannabis use was more common among individuals who smoked prenatally (PRR = 3.84, 95% CI = 2.55–5.78) and among those who used alcohol during pregnancy (PRR = 1.80, 95% CI = 1.25–2.59). In this bivariate analysis, lower levels of education and income, and being unmarried, were the strongest correlates of cannabis use in pregnancy.

**TABLE 2:** Maternal characteristics by prenatal cannabis use status in Michigan, 2017–2023.

	Total population	Cannabis use during pregnancy	
	*n* ^a^ (weighted %)	*n* ^a^ (weighted %)	PRR^b^ (95% CI)
**Socio‐demographic status**			
Maternal age			
<25 years	230 (22.2%)	85 (30.3%)	**3.13 (1.52–6.42)**
25–29 years	306 (30.9%)	72 (16.5%)	**1.70 (1.10–2.63)**
≥30 years	463 (47.0%)	69 (9.7%)	1 (ref.)
Race/ethnicity^c^			
Non‐Hispanic White	619 (68.3%)	89 (10.6%)	1 (ref.)
Non‐Hispanic Black	285 (17.0%)	115 (41.9%)	**3.95 (2.58–6.05)**
Other^d^	91 (14.7%)	19 (12.8%)	1.21 (0.55–2.69)
Marital status			
Married	503 (60.5%)	32 (4.1%)	1 (ref.)
Not married	494 (39.5%)	192 (34.9%)	**8.54 (5.49–13.29)**
Income			
≤$24,999	343 (26.1%)	147 (40.5%)	**12.81 (6.11–26.85)**
$25,000–$74,999	257 (30.6%)	54 (13.0%)	**4.10 (2.13–7.89)**
≥$75,000	361 (43.3%)	10 (3.2%)	1 (ref.)
Education			
High school or below	292 (25.1%)	124 (34.5%)	**16.69 (6.01–46.33)**
Some college	346 (35.0%)	91 (19.6%)	**9.46 (3.06–29.29)**
Undergraduate or above	358 (39.9%)	9 (2.1%)	1 (ref.)
Enrollment in government health plan			
No	514 (59.8%)	43 (6.5%)	1 (ref.)
Yes	474 (40.2%)	178 (30.3%)	**4.64 (2.33–9.24)**
**Psychological factors**			
Pregnancy intention			
Unplanned	486 (44.2%)	163 (26.0%)	**3.10 (1.91–5.05)**
Planned	496 (55.8%)	56 (8.4%)	1 (ref.)
Symptoms of depression (EDS ≥ 12)			
No	777 (85.9%)	144 (13.1%)	1 (ref.)
Yes	159 (14.1%)	74 (40.6%)	**3.10 (2.22–4.34)**
General social support (median)			
≤61	531 (49.3%)	153 (22.6%)	**2.20 (1.44–3.35)**
>61	459 (50.7%)	70 (10.3%)	1 (ref.)
Adverse childhood experiences (ACEs)			
0 ACEs	471 (51.7%)	51 (7.1%)	1 (ref.)
1–2 ACEs	352 (34.1%)	102 (21.1%)	**2.95 (1.71–5.08)**
≥3 ACEs	142 (14.2%)	60 (35.2%)	**4.93 (2.64–9.21)**
Experiences of discrimination			
0 experiences	774 (80.6%)	145 (13.0%)	1 (ref.)
1–2 experiences	127 (11.5%)	41 (24.4%)	**1.88 (1.36–2.64)**
≥3 experiences	88 (7.9%)	35 (38.1%)	**2.94 (1.73–4.99)**
**Other substance use**			
Tobacco smoking during pregnancy			
No	760 (83.5%)	108 (11.1%)	1 (ref.)
Yes	238 (16.5%)	118 (42.8%)	**3.84 (2.55–5.78)**
Alcohol use during pregnancy			
No	865 (88.8%)	186 (15.0%)	1 (ref.)
Yes	129 (11.2%)	38 (26.9%)	**1.80 (1.25–2.59)**

Abbreviations: ACEs, adverse childhood experiences; EDS, Edinburgh Depression Scale; PRR, prevalence rate ratio.

^a^ Unweighted sample size.

^b^ PRR was calculated using Poisson regression with a log‐link function and robust standard errors accounting for the sampling weights and clustering by sampled hospital.

^c^ We adopt Buchanan *et al*.’s [34] reporting of race as ‘a political and social construct that often serves as a proxy for the impact of racist practices and structural inequality, it is not a biological variable and thus is examined in the current paper with this premise in mind’.

^d^ Other includes the following weighted percentages based on the overall sample included in this analysis: 8.0% Hispanic, 1.9% Mixed race, 1.9% Asian Indian, 1.4% Asian, and less than 1% for Chinese, Korean, Japanese and other races.

**FIGURE 3:**
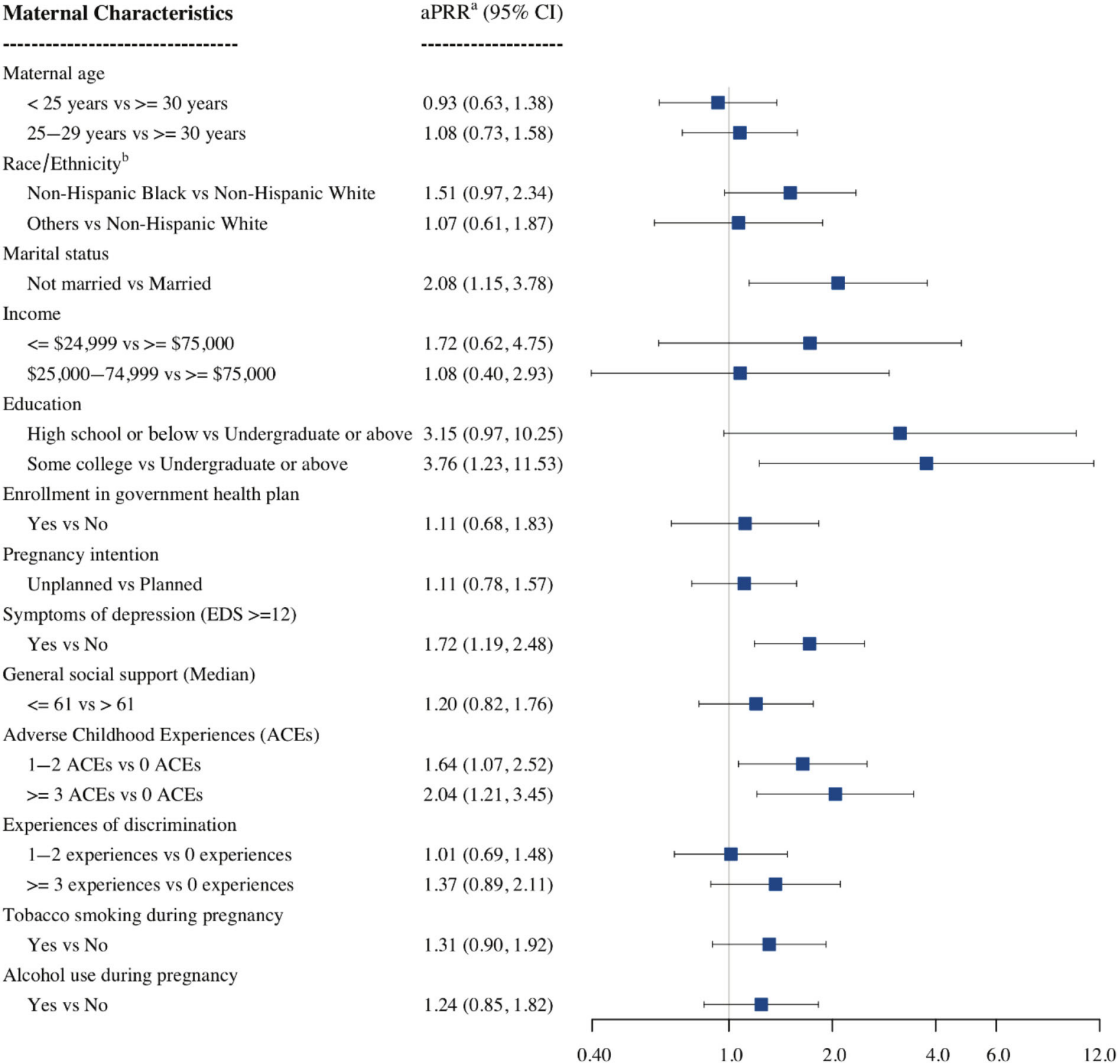
Adjusted associations between maternal characteristics and prenatal cannabis use in Michigan, 2017–2023.

The multivariate analysis of the characteristics of prenatal cannabis use is depicted in Figure 3. Education, marital status, symptoms of depression and ACEs remained significantly associated with cannabis use, after adjusting for all covariates. Unmarried participants used cannabis during pregnancy twice as often as married participants (adjusted PRR, aPRR = 2.08, 95% CI = 1.15–3.78). Compared with participants with an undergraduate degree or higher, the prevalence of cannabis use was 3.76 times (95% CI = 1.23–11.53) and 3.15 times (95% CI = 0.97–10.25) more for participants with some college education and for participants without any college education, respectively. Participants who reported depressive symptoms were 1.72 times (95% CI = 1.19–2.48) more likely to use cannabis than participants with no depressive symptoms. A history of ACEs showed a dose–response relationship with prenatal cannabis use.

## DISCUSSION

The present study provides a unique contribution in estimating the prevalence and associated characteristics of cannabis use via self‐report and urine toxicology testing during pregnancy in a stratified random sample of an entire state. Our results suggest that around 25% of the participants self‐reported cannabis use 3 months before pregnancy, and almost half of them (12.3%) self‐reported continued use during pregnancy. When urine toxicology measures (13.3%) were considered, the weighted prevalence of cannabis use in pregnancy increased to 16.8%, or approximately one in six pregnancies in Michigan as a whole, and as many as one in four pregnancies in some parts of the state. Although the rates of self‐reported cannabis use and urinalysis seem congruent, more than half of prenatal cannabis use was detected by only one of the ascertainment methods, contributing to the higher overall prevalence rate. Participants who were single (aPRR = 2.08, 95% CI = 1.15–3.78), had lower educational attainment (aPRR = 3.76, 95% CI = 1.23–11.53, for some college education vs undergraduate education or above; aPRR = 3.15, 95% CI = 0.97–10.25, for high school education or below vs undergraduate education or above), reported symptoms of depression (aPRR = 1.72, 95% CI = 1.19–2.48) and had a history of adverse childhood events (aPRR = 2.04, 95% CI = 1.21–3.45, for ≥3 ACEs vs 0 ACES; aPRR = 1.64, 95% CI = 1.07–2.52, for 1–2 ACEs vs 0 ACEs) were more likely to use cannabis during pregnancy.

In the absence of national and statewide cannabis prevalence estimates validated by toxicology testing, our results can only be compared with studies conducted on pregnant populations within specific healthcare systems or geographical areas. Among the few studies that used both self‐reports and urine toxicology to assess prenatal cannabis use, a study in Northern California among insured Kaiser Permanente patients found a prevalence of 9.0% (95% CI = 8.7%–9.2%) in 2022 [35], whereas a hospital‐based study in a single tertiary center in Colorado during 2011–2017 reported a prevalence of 17.5% [36].

Considering only self‐reported cannabis use, our findings are generally higher than those reported from international and national data. The prevalence of cannabis use in a large study conducted in Canada, which federally legalized cannabis around the same time as Michigan, was reported to be 2.5% in 2018–2019 [37]. In the USA, on the other hand, past‐month cannabis use was reported in 4.9% of pregnancies according to the most recent data (2013–2019) from the National Survey on Drug Use and Health (NSDUH) [38]. In comparison with other states, the MARCH self‐reported rate is also considerably higher than in most states with and without recreational cannabis laws. Data from the 2017 cycle of the Pregnancy Risk Assessment Monitoring System (PRAMS), a population‐based surveillance system managed by the Centers for Disease Control and Prevention (CDC) that interviews during the postpartum period, found prevalences of 2.6% in New York State [39] (before the legalization of recreational cannabis), 7.2% in Colorado [40] and 12.1% in Maine [39] (where recreational cannabis was legal in both states). The PRAMS and MARCH findings in Michigan, however, are discrepant. The PRAMS findings suggest an average weighted prevalence rate for prenatal cannabis use of around 7.0% between 2017 and 2022, the same time period as our study [41]. This difference may stem, at least in part, from the retrospective collection of data in PRAMS, which interviews mothers between 11 weeks and 9 months postpartum, whereas MARCH collected cannabis use data in real time during pregnancy [33].

The high prevalence of cannabis use detected among pregnant individuals in Michigan reflects the interplay of many factors, including safety perception, drug availability and local cannabis policies. Based on NSDUH data (2015–2017), one‐fifth of pregnant women (21.6%, 95% CI = 19.4%–23.8%) believed that weekly cannabis use is without risk [5]. With expanded legalization, these safety perceptions are increasing among both pregnant and non‐pregnant female populations in the USA [4, 6]. In an outpatient clinic in Maryland, at a time when only medical cannabis was legal, 62% of women using cannabis suggested that they might increase their intake during pregnancy if recreational cannabis was legalized [42]. Pregnant individuals, based on qualitative studies, believe that cannabis is a ‘natural’ remedy that might be ‘safer’ and ‘less harmful’ than prescription drugs [43, 44]. Participants who report cannabis use mainly use it to relieve stress or anxiety (81.5%), nausea and vomiting (77.8%), and pain (55.1%) [39, 44]. Our group has previously shown, using a subset of the MARCH data, that the severity of morning sickness slightly increased the odds of using cannabis (OR = 1.2; 95% CI = 1.1–1.2) [45]. Moreover, a study using NSDUH data reported that 91.7% of pregnant women who self‐reported cannabis use in the past year found it fairly or very easy to acquire cannabis [46]. In Michigan, specifically, cannabis products are becoming more available and affordable since legalization. The cannabis retail market in Michigan might have lower barriers to entry and it is currently among the most dominant markets in the USA by sales volume, surpassing California [47]. The average cannabis retail flower price has also decreased from $267.30 per ounce in December 2019 to $65.14 per ounce in March 2025 [48]. Although recreational cannabis is legal in Michigan, substate jurisdictions have the authority to opt in or out of having retail dispensaries. Substate regions with the highest sales of cannabis products in 2025, East Michigan and Metro Detroit, were also the same regions with the highest prevalence of prenatal cannabis use observed in our study [48]. Young‐Wolff *et al*. (2022) revealed that cannabis use during pregnancy was higher in areas that had more cannabis retail outlets in California [49].

Consistently, cannabis use among adults in Michigan also seems to be higher than in the USA overall as well as in the Midwest region [50]. Adult use in Michigan was also highest in substate regions similar to those in our study [51]. Although research suggests an association between recreational cannabis legalization and increased cannabis use among adults [52, 53, 54, 55], the effects of legislation on the pregnant population is uncertain [56, 57]. Further research is needed to better understand how the legal status of cannabis is related to the high pregnancy prevalence we found. The findings should also prompt clinicians to screen for its use early during pregnancy. This may help to create an open dialog with pregnant patients on cannabis use cessation and harm reduction strategies. It may also help providers to understand the specific needs of their patients to provide trauma‐informed and culturally responsive counselling that is based on respect, dignity and self‐autonomy.

The characteristics of pregnant individuals using cannabis during pregnancy in our study were consistent with prior literature. Both Sood *et al*. [58] and Ko *et al*. [39] reported elevated prenatal cannabis use among unmarried women, and the latter study also found higher cannabis use with lower educational achievement. A national study among pregnant and postpartum women found substantially higher past‐year cannabis use in women reporting depressive disorders [59], a finding replicated in Kaiser Permanente pregnant patient data [60]. Although depression might lead to cannabis use, it is also plausible that individuals with mental health issues may self‐medicate with cannabis. In a number of states, mental health issues, including depression, are qualifying conditions for medical cannabis [61]. Both Thomas *et al*. [62] in Nevada and Testa *et al*. [63] in North and South Dakota found dose–response relationships between cannabis use and the number of ACEs reported by pregnant individuals. Study findings highlight the importance of screening for ACEs and symptoms of depression during prenatal care visits to help mitigate their potential effect on cannabis use and their synergistic impact on adverse birth outcomes.

Although significant at the bivariate level, maternal age, income, pregnancy intention, enrollment in government health plan and race/ethnicity were not significantly associated with cannabis use in the adjusted model. The results are in agreement with the literature, where maternal age [39, 58, 64], health insurance [39] and pregnancy intention [58] also failed to remain significant in the multivariate analysis. Marital status in our study as well as in the literature seems to have the strongest association with cannabis use, compared with other socio‐demographic variables. A partner’s support, health behaviors and substance use practices may have an influence on cannabis use during pregnancy. Based on the study findings, it appears that ACEs or exposure to traumatic life events during childhood, which are disproportionally experienced by individuals of specific races/ethnicities and economic backgrounds, might explain more variation in the prevalence of cannabis use than the demographic variables themselves.

This study has some limitations. Because the cohort was not designed solely to investigate cannabis use, we did not obtain details on the frequency and mode of use, use by trimester, reasons for use and postnatal practices, all of which would have added further insight. In addition, we had no measurements from questionnaires or urinalysis for cannabidiol (CBD). The longer duration of detectability of urinary THC among individuals with heavy and frequent cannabis use than in those with occasional use [65] suggests that our urine assays preferentially detected such individuals while underestimating cannabis use among those with occasional use. Moreover, because the annual recruitment of participants during the study period varied by clinic, we could not assess changes in use over time, nor securely link our findings to dates of legalization of cannabis in Michigan, given that there were large differences in the rates of cannabis use by hospital, and it is unclear whether any time trends would arise from overall population changes or differences in the sampled hospitals at a given point in time.

## CONCLUSION

Our study aimed to examine, using validated and objective measures, the statewide prevalence of cannabis use in Michigan in the era of increasing legalization of cannabis. Despite recommendations against using cannabis during pregnancy by the American College of Obstetricians and Gynecologists [65], we estimate that cannabis exposure occurs in at least one in six pregnancies in Michigan. Cannabis use was more common among socially disadvantaged populations and among individuals with depressive symptoms and histories of ACEs. Providing equitable and compassionate patient‐centered counseling to inform decisions on cannabis use in pregnancy is warranted.

## AUTHOR CONTRIBUTIONS

**Ban Al‐Sahab:** Conceptualization (lead); data curation (lead); formal analysis (lead); funding acquisition (lead); investigation (lead); methodology (lead); project administration (lead); visualization (lead); writing—original draft (lead); writing—review and editing (equal). **Jean M. Kerver:** Conceptualization (equal); funding acquisition (lead); investigation (equal); methodology (equal); supervision (lead); writing—review and editing (equal). **Omayma Alshaarawy:** Conceptualization (equal); data curation (equal); methodology (equal); supervision (equal); writing—review and editing (equal). **Kipling M. Bohnert:** Conceptualization (equal); methodology (equal); supervision (equal); writing—review and editing (equal). **Michael R. Elliott:** Conceptualization (equal); data curation (equal); formal analysis (equal); funding acquisition (equal); methodology (equal); supervision (equal); writing—review and editing (equal). **Hongxiang Qiu:** Formal analysis (equal); methodology (equal); software; visualization (equal); writing—review and editing (equal). **Audriyana Jaber:** Data curation (equal); methodology (equal); project administration (equal); resources (equal); writing—review and editing (equal). **Harish Neelam:** Formal analysis (equal); methodology (equal); software (equal); visualization (equal); writing—review and editing (equal). **Nigel Paneth:** Conceptualization (equal); funding acquisition (lead); investigation (lead); methodology (equal); supervision (lead); writing—review and editing (equal).

## DECLARATION OF INTERESTS

All authors report no conflicts of interest.

## Supporting information


**Appendix S1.** Supporting information.

**Figure S1.** Determination of study population for each objective.

## Data Availability

Deidentified data is available upon request.
