# Supplementary material for: Prevalence and characteristics of prenatal cannabis use in Michigan, USA: A statewide population‐based pregnancy cohort
Source: Addiction. 2025 Sep 15;121(1):126–37. doi: 10.1111/add.70188 (PMC12710835; doi:10.1111/add.70188)
Supplement: Supplementary file 1 — Appendix S1. Supporting information. [file ADD-121-126-s001.docx]

**Supplemental Methods: MARCH study design**

The Michigan Archive for Research on Child Health (MARCH) cohort was a prospective statewide pregnancy cohort based on a stratified random sample of births in the 95 hospitals with maternity services in the lower peninsula of Michigan, where 97% of Michigan hospital births take place (1, 2). Hospitals were stratified into five quintiles based on fraction of births to African American individuals, to ensure representation both of large metropolitan areas and small rural settings. Two hospitals were randomly chosen from each quintile using proportional-to-size sampling, yielding 10 hospitals for the study. All 10 sampled hospitals agreed to participate, yielding a first-stage response rate of 100%. Prenatal clinics referring to these 10 hospitals were also sampled proportional-to-size and two clinics were selected for each hospital; 13 clinics agreed to participate (65% second-stage response rate). Six out of the 7 clinics that refused participation were replaced. One clinic that did not participate could not be replaced because only two clinics served the selected hospital. Recruitment in two clinics did not yield the required number of participants and were also later replaced. An additional hospital in Flint and its sole prenatal care clinic were added to the study outside the probability sampling design. Overall, recruitment was primarily conducted in 20 clinics serving 11 hospitals across the state, with partial recruitment from 2 additional clinics.

Pregnancies were recruited in 2017-2023 at the first prenatal visit of all consenting patients over age 18 years who could complete a survey in English. In order to approximate an equal probability of selection for births, a recruitment total of 100 births per hospital was established, and 1,130 births were obtained from 1,105 recruited pregnancies (there were 25 pregnancies resulting in twin births). A minimum of 306 persons were approached for the study but refused to participate, yielding an upper bound third-stage response rate of 78.7% or a total upper bound response rate of 51.2%. Sampling weights were constructed to account for the differences in the hospital size measures (number of births) at the design stage and the actual data collection period, and to adjust the distribution of the MARCH sample using raking to the known population distribution of mother’s age, race/ethnicity, education level, marital status, gestational age, self-reported use of cigarettes and alcohol, and gestational diabetes, and child sex, birthweight, and Apgar score obtained from Michigan birth certificate data (3). Detailed information about the MARCH cohort is available elsewhere (4, 5).

**References:**

1. MDHHS. Live Births by County of Residence, State of Michigan, Michigan Counties and Detroit City, 2012 - 2022 [cited 2025 January]. Available from: <https://www.mdch.state.mi.us/osr/natality/BirthsTrends.asp>.

2. Lang G, Farnell EAt, Quinlan JD. Out-of-Hospital Birth. Am Fam Physician. 2021;103(11):672-9.

3. Bishop YM, Light RJ, Fienberg SE, Mosteller F, Holland PW. Discrete Multivariate Analysis: Theory and Practice: Springer New York; 2007.

4. Elliott MR KJ, Drew A, Watson K, Kornatowski B, Norman GS, Copeland GE, Leissou E, Ridenour T, Kruger-Ndiaye S, Ma T, Ruden D, Barone C, Keating DP, Sokol RJ, Johnson CC, Paneth N. Obtaining a Probability Sample of a Pregnancy Cohort of Births: A Review of the Problem and a Practical Solution. Am J Epidemiol. *In press*.

5. CHARM. Child Health Advances from Research with Mothers [cited 2024 November]. Available from: <https://charmstudy.epibio.msu.edu/>.
