# Supplementary material for: Prevalence and characteristics of prenatal cannabis use in Michigan, USA: A statewide population‐based pregnancy cohort
Source: Addiction. 2025 Sep 15;121(1):126–37. doi: 10.1111/add.70188 (PMC12710835; doi:10.1111/add.70188)
Supplement: Supplementary file 2 — Figure S1. Determination of study population for each objective. [file ADD-121-126-s002.docx]

**Supplemental Figure:** **Determination of study population for each objective**

**1,105**  Eligible pregnant participant who consented to MARCH Cohort and gave birth at sampled hospitals

**13**  Missing cannabis data

**10** Missing prenatal survey & urine samples

**2** Missing prenatal survey & urine aliquot size less than 3 ml

**1** Missing prenatal survey & creatinine levels below human levels

**1,092**  Included in analysis for objective 1

**93**  Missing Prenatal 1 survey data

**999** Included in analysis for objective 2
